# Alterations in blood flow at the optic nerve head in patients with thyroid eye disease using optic coherence tomography angiography

**DOI:** 10.3389/fmed.2025.1585907

**Published:** 2025-05-30

**Authors:** Shenglan Cao, Rongfeng Liao, Chengbo Fang

**Affiliations:** Department of Ophthalmology, The First Affiliated Hospital of Anhui Medical University, Hefei, China

**Keywords:** thyroid eye disease, peripapillary vessel density, retinal nerve fiber layer thickness, optic coherence tomography angiography, radial peripapillary capillaries

## Abstract

**Introduction:**

This study aimed to evaluate changes in blood flow at the optic nerve head (ONH) in patients with thyroid eye disease (TED) using optical coherence tomography angiography (OCTA).

**Methods:**

A total of 59 eyes from 59 patients with TED and 39 eyes from 39 healthy controls (HCs) were included. Patients with TED were categorized into the active and inactive groups based on clinical activity scores. All participants underwent ophthalmological and endocrinological tests. Peripapillary vessel density (VD) and retinal nerve fiber layer thickness (RNFLT) were measured using OCTA. All eyes underwent a 4.5*4.5-mm scan pattern centered on the ONH. The peripapillary region was divided into eight sectors: superior nasal (SN), nasal upper (NU), nasal lower (NL), inferior nasal (IN), inferior temporal (IT), temporal lower (TL), temporal upper (TU), and superior temporal (ST). A correlation analysis was used to explore the association between blood flow changes and relevant factors.

**Results:**

Compared to the eyes of HCs, the eyes of patients in the inactive TED group demonstrated lower radial peripapillary capillary VD, with significant differences observed in the SN and ST sectors of the peripapillary region (*p* < 0.05). No differences were observed in the peripapillary RNFLT between the groups. Additionally, there was no significant correlation between the whole peripapillary VD and RNFLT or visual functional parameters. Moreover, visual acuity impairment was positively correlated with IN-VD, NU-VD, ST-VD, and NU-RNFLT in the peripapillary region. Intraocular pressure was positively correlated with IN-RNFLT.

**Conclusion:**

OCTA can detect microvascular ONH changes in patients with TED.

## 1 Introduction

Thyroid eye disease (TED) is a well-known autoimmune disorder and a potentially sight-threatening ocular disease (1, 2). It generally occurs in patients with hyperthyroidism, euthyroidism, or chronic hypothyroid autoimmune thyroiditis. The pathophysiology of TED is not completely clear; however, it is characterized by inflammation, adipogenesis, and glycosaminoglycan accumulation. It has been suggested that orbital fibrocytes are the primary factor in TED, with the activation of orbital fibroblasts by thyrotrophin receptor antibody binding leading to the fibrosis of extracellular muscles and deposition of glycosaminoglycans, resulting in orbital tissue remodeling (3, 4). In addition, few studies have focused on the role of autophagy in the development of TED, and abnormal autophagy regulation can lead to orbital inflammation, adipogenesis, and glycosaminoglycan accumulation (5). The most common clinical features of TED are upper eyelid retraction, edema, proptosis, conjunctivae, dysfunctional eye movements, and erythema around the periorbital tissue (6, 7). A few patients with TED (nearly 4–8%) experience severe features, such as corneal ulceration and dysthyroid optic neuropathy (1, 8, 9). However, diagnosing dysthyroid optic neuropathy is challenging until irreversible and profound visual loss occurs. Previous studies have shown that ocular perfusion fluctuates during different stages of TED and that dysthyroid optic neuropathy is associated with reduced perfusion (10). Using color Doppler sonography, it was observed that decreased blood flow velocity in the superior ophthalmic vein (SOV), possibly due to the compression of the vein at the apex of the orbit, causes venous stasis (11).

In recent years, optical coherence tomography angiography (OCTA), a safer, non-invasive, and novel angiographic technique, has shown the microcirculation of the retina and optic nerve head (ONH) in a three-dimensional (3D) manner, unlike fluorescein or indocyanine green angiography (12). OCTA has been widely used to analyze vessel density (VD) changes in the radial peripapillary capillary (RPC) and retinal nerve fiber layer thickness (RNFLT) in the ONH in patients with glaucoma, diabetic retinopathy, and ischemic optic neuropathy (13–16). However, its use in TED is limited. OCTA can provide a quick and sensitive analysis of the perfusion of RPCs; therefore, it might be useful in monitoring the very early stages of TED before irreversible visual impairment. As the superficial layer of capillaries, the RPCs lie in the inner part of the RNFL and nourish the inner portion of the RNFL around the ONH (17). Studies (18, 19) have reported that RPCs play an important role in metabolism and function in both health and disease, such as glaucoma and ischemic optic neuropathy, and are useful for the early prediction of the progression of optic neuropathy diseases.

This study aimed to use OCTA to identify and quantitatively analyze blood flow changes in the ONH of patients with TED at different stages and provide a more specific assessment of visual function and progression in TED.

## 2 Materials and methods

A total of 59 eyes from 59 patients with TED and 39 eyes from 39 healthy controls (HCs) were included in this study. The patients were recruited between June 2020 and July 2024. Patients with TED were diagnosed by an endocrinologist and an ophthalmologist at the First Affiliated Hospital of Anhui Medical University. The patients were divided into two groups (active and inactive) based on their clinical activity scores (CAS): (1) spontaneous orbital pain, (2) staring-induced orbital pain, (3) eyelid swelling, (4) eyelid erythema, (5) conjunctival redness, (6) suppurative necrosis, and (7) sarcoma inflammation or plica. A CAS of ≥3/7 indicated active disease, while a score of < 3/7 indicated inactivity.

Age- and sex-matched healthy controls, such as companions of patients or hospital staff, were enrolled among individuals without ocular or systemic diseases. The study was performed in accordance with the tenets of the Declaration of Helsinki and adhered to the requirements of the ethics committee of the First Affiliated Hospital of Anhui Medical University (PJ-2024-11-74). The exclusion criteria were as follows: (1) other ocular diseases, such as cataracts, glaucoma, uveitis, or retinal disease; (2) spherical equivalent of ≥ 3.00D; (3) steroid pulse therapy or local radiotherapy within 3 months; (4) a history of ocular surgery; (5) ocular trauma; and (6) other severe systemic diseases, such as diabetes and hypertension.

All participants underwent complete ocular examinations, including best-corrected visual acuity (BCVA) measured using an international standard logarithmic visual acuity chart, slit lamp biomicroscopy, intraocular pressure (IOP, mmHg) measured by tonometry, ocular proptosis evaluation performed using a Hertel exophthalmometer, and fundus examination using biomicroscopy. The age, sex, duration of thyroid disease, smoking status, therapy history, and laboratory tests, such as the thyroid stimulating hormone (TSH) level, of the patients were recorded in detail.

### 2.1 OCTA

**Figure 1:**
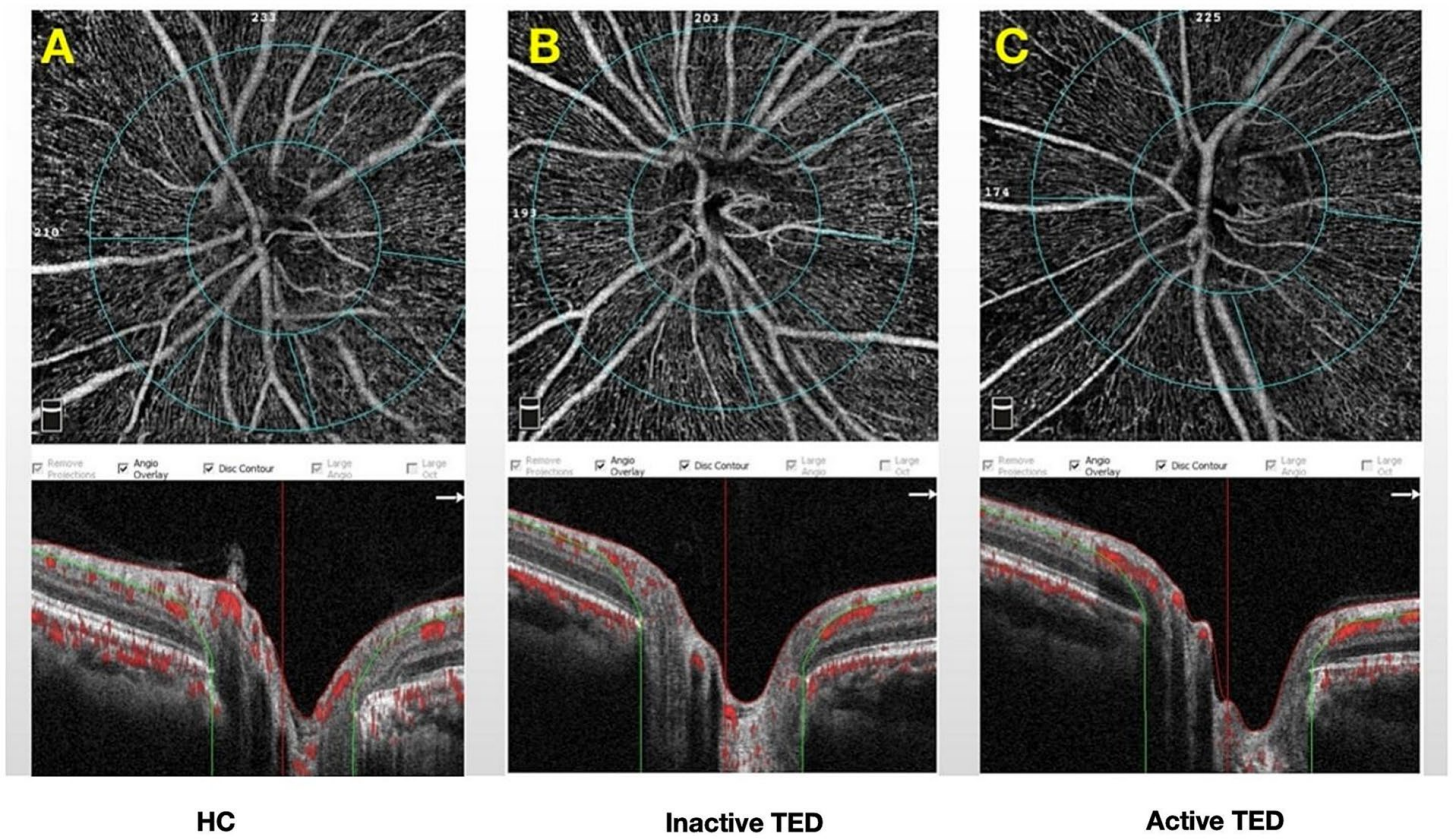
Measurement of optical coherence tomography angiography parameters centered on the optic nerve head with a 4.5*4.5-mm rectangle scan. The peripapillary region was defined by the software as a 1.0-mm wide round annulus extending from the optic disc boundary. Peripapillary maps from eyes representative of **(A)** healthy control (HC) and **(B)** inactive and **(C)** active thyroid eye disease (TED) groups.

**Figure 2:**
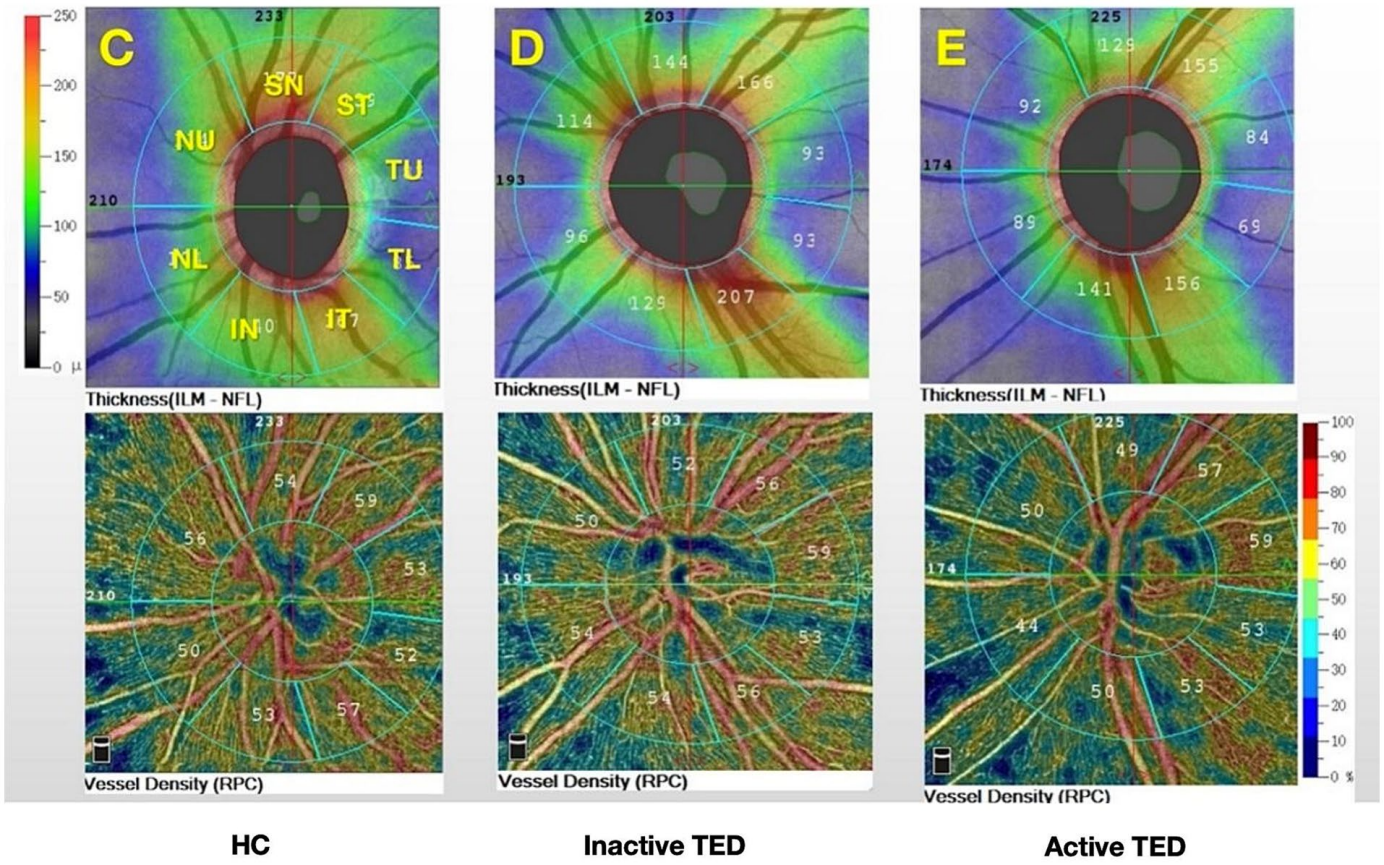
Color maps show the retinal nerve fiber layer thickness and vessel density for the eight sectors of the peripapillary region based on the modified Garway–Heath map **(C–E)**: superior nasal (SN); nasal upper (NU); nasal lower (NL); inferior nasal (IN); inferior temporal (IT); temporal lower (TL); temporal upper (TU); and superior temporal (ST) and from **(C)** healthy controls (HCs) and **(D)** inactive and **(E)** active thyroid eye disease (TED) groups.

OCTA of the optic disc was performed using the AngioVue OCTA System (Optovue, Inc., Fremont, California, USA). The scanning speed was 70,000 A-scans/s, and the wavelength was 840 nm. The ONH was scanned using a 4.5*4.5-mm scan pattern. The newly developed, built-in Angio Analytics software (version 2017.1.0.151; Optovue, Inc.) was used to evaluate peripapillary VD and RNFLT. The peripapillary region was defined by the software as a 1.0-mm wide round annulus extending from the optic disc boundary. The default quantified vascular layer is the superficial retinal layer, which extends from the inner membrane to the posterior boundary of the retinal nerve fiber layer (Figure 1). Based on the modified Garway–Heath map (20), the clinical map is valuable in the clinical diagnosis of glaucoma and the research of ONH structure. The peripapillary region was further divided into eight sectors: superior nasal (SN), nasal upper (NU), nasal lower (NL), inferior nasal (IN), inferior temporal (IT), temporal lower (TL), temporal upper (TU), and superior temporal (ST) (Figure 2). Based on the CAS classification, the less severe eye of each patient was selected. When the two eyes from a patient had the same severity, the eye with a higher scan quality index was chosen. In addition, only signal strengths of >5 were included in the analysis.

### 2.2 Statistical analysis

All analyses were performed using SPSS software for Windows (version 23.0; IBM Corp., Armonk, NY, USA). Continuous variables were expressed as mean ± standard deviation. A one-way analysis of variance (ANOVA) was used to compare differences in peripapillary VD and RNFLT among the groups, and the LSD method was used for *post-hoc* comparisons. The studied parameters were compared between groups using the χ^2^ test and the independent samples *t*-test, such as sex, duration, TSH level, proptosis, and IOP values in patients with TED between the active and inactive groups. Pearson’s correlation analysis was performed to assess the relationships between the VD, RNFLT, and visual function parameters. A *p*-value of <0.05 was considered statistically significant.

## 3 Results

### 3.1 Demographics and clinical characteristics

**Figure 3:**
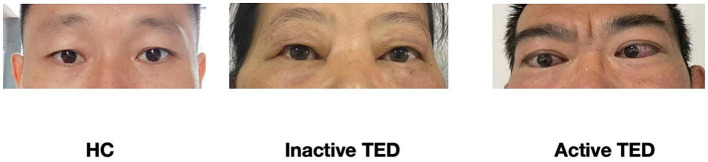
Clinical photograph of three different groups of participants.

In total, 59 eyes from 59 patients with TED (33 active, CAS ≥ 3; 26 inactive, CAS < 3; 25 men; 34 women) and 39 eyes from 39 healthy controls (HCs) (20 men and 19 women) were enrolled in the present study (Figure 3). The demographic data of all participants are presented in Table 1. However, there were no intergroup differences in age or sex distribution. Patients with TED had a mean duration of ocular signs of 10.10 ± 13.06 months. In addition, 30.90% of patients had received steroid pulse therapy, 6.78% had received orbital radiotherapy, and 23.73% were smokers.

**Table 1:** Demographics and clinical characteristics of all participants.

Parameters	Active TED patients	Inactive TED patients	HCs	*χ*^2^/*F*/*t* value	*p* value
Patients, *n*	33	26	39		
Sex (M: F)	15:18	10:16	20:19	1.037^a^	0.595
Age, years	44.76 ± 8.47	40.08 ± 10.78	44.38 ± 9.98	2.039^b^	0.136
Exophthalmos (mm)	18.72 ± 3.15	18.34 ± 2.15	NA	1.339^c^	0.180
IOP (mmHg)	17.3 ± 3.56	18.46 ± 4.63	13.18 ± 1.92	24.505^b^	0.000
Duration, months	9.49 ± 12.92	10.12 ± 14.33	NA	-0.178^c^	0.859
TSH (μIU/ml)	3.71 ± 7.54	2.99 ± 4.14	NA	0.442^c^	0.660
BCVA, logMAR	0.29 ± 0.30	0.18 ± 0.24	0.04 ± 0.07	11.775^b^	0.010

TED, thyroid eye disease; HCs, healthy controls; M, male; F, female; BCVA, best-corrected visual acuity; IOP, intraocular pressure; NA, not applicable; a *p*-value of <0.05 is considered statistically significant. a, *χ*^2^ value; b, *F*-value; c, *t*-value.

Patients with TED had significantly lower BCVA (mean: 0.29 ± 0.30, 0.18 ± 0.24 in the active and inactive groups, respectively) than the HCs (mean: 0.04 ± 0.75). Mean IOP values were 17.3 ± 3.56 and 18.46 ± 4.63, which were significantly higher than the HCs (mean: 13.18 ± 1.92). Mean proptosis values were 18.72 ± 3.15 and 18.34 ± 2.15 in the active and inactive groups, respectively.

### 3.2 Analysis of peripapillary VD and RNFLT

**Figure 4:**
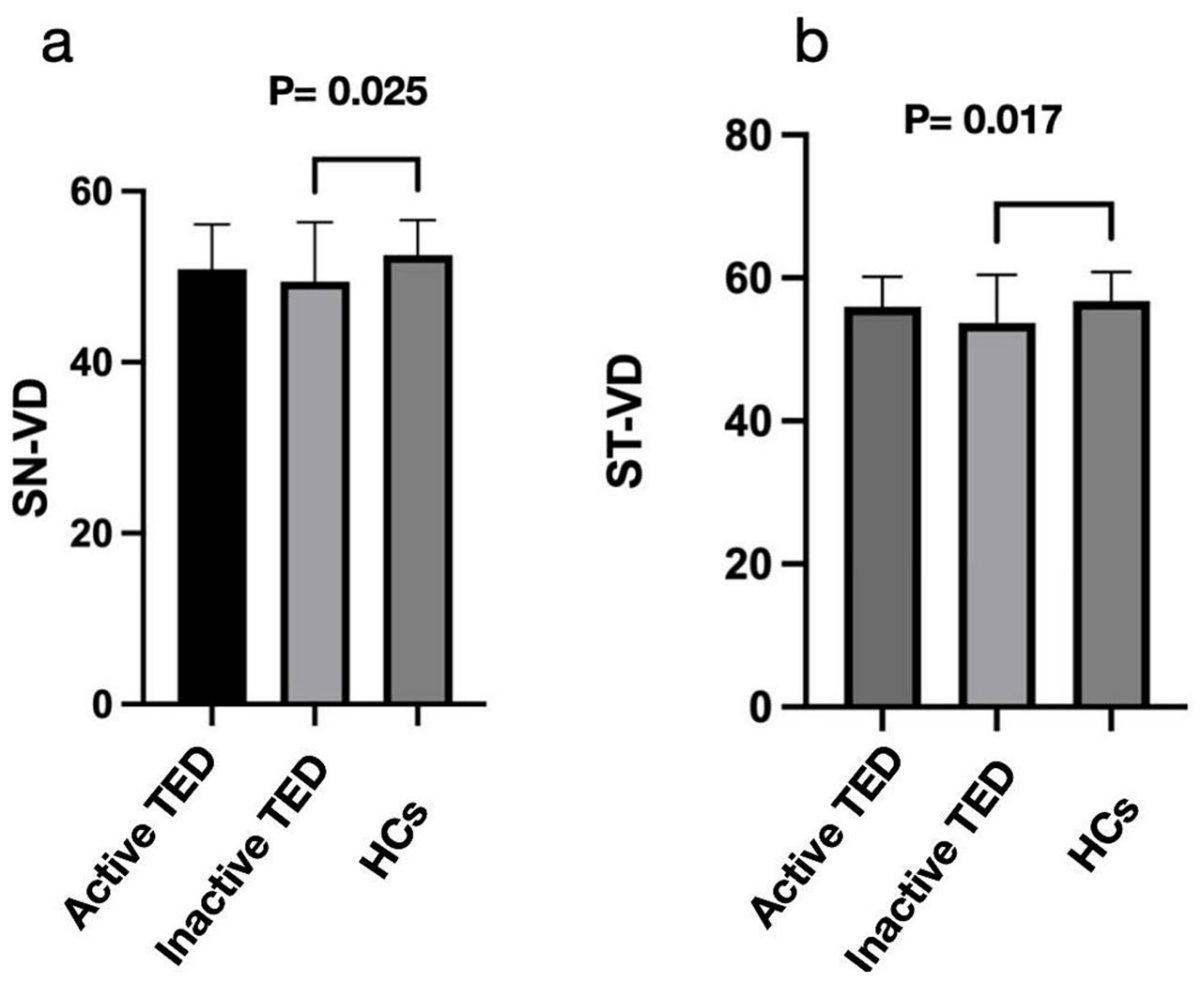
Comparisons of the vessel density (VD) and retinal nerve fiber layer thickness on optical coherence tomography angiography images between the three groups in the eight sectors. The superior nasal (SN) and superior temporal (ST) sectors of peripapillary VD in the inactive thyroid eye disease (TED) patients showed significant differences with the corresponding values in the healthy control (HC) group **(a,b)**.

Comparison of the peripapillary ONH-VD and peripapillary VD of the eight sectors between the study group and HCs using OCTA did not reveal any significant differences in ONH-VD measurements between the groups. However, the measurements of the SN and ST sectors of the peripapillary VD in the inactive group were significantly different from those of the HCs (*p* = 0.025 and 0.017, respectively) (Table 2, Figure 4). Furthermore, there were no significant differences in the mean RNFLT of the peripapillary region between the patients with TED and HCs in all eight sectors (all *p* > 0.05) (Table 3).

**Table 2:** Comparison of peripapillary vessel density between three groups.

Region	Active TED patients *n* = 33	Inactive TED patients *n* = 26	HCs *n* = 39	*F* value	*Post-hoc* analysis *p* value		
					A VS I	A VS H	I VS H
Peri-VD	52.17 ± 2.76	51.92 ± 4.74	53.48 ± 2.24	2.325	0.766	0.089	0.059
SN	50.88 ± 5.26	49.42 ± 6.94	52.51 ± 4.12	2.648	0.303	0.201	0.025*
NU	48.64 ± 4.07	50.12 ± 14.33	50.03 ± 4.67	0.732	0.394	0.247	0.841
NL	46.79 ± 5.82	47.96 ± 5.51	48.33 ± 4.68	0.769	0.401	0.221	0.783
IN	52.06 ± 4.24	52.04 ± 6.27	53.95 ± 4.36	1.760	0.986	0.107	0.127
IT	58.30 ± 5.02	57.20 ± 6.74	59.31 ± 3.93	1.322	0.414	0.412	0.109
TL	52.70 ± 4.39	51.92 ± 5.73	54.00 ± 4.52	1.549	0.542	0.257	0.092
TU	55.67 ± 5.27	55.96 ± 4.55	55.76 ± 9.75	0.012	0.877	0.953	0.917
ST	55.94 ± 4.20	53.73 ± 6.73	56.77 ± 4.07	3.005	0.092	0.480	0.017*

A, active group; I, inactive group; H, healthy controls *, a *p*-value of <0.05 is considered statistically significant.

**Table 3:** Comparison of peripapillary retinal nerve fiber layer thickness between three groups.

Region	Active TED patients *n* = 33	Inactive TED patients *n* = 26	HCs *n* = 39	*F* value	*Post-hoc* analysis *p* value		
					A VS I	A VS H	I VS H
Peri-RNFLT	117.00 ± 19.05	118.26 ± 19.32	119.69 ± 9.53	0.254	0.830	0.334	0.262
SN	140.30 ± 34.93	138.69 ± 28.15	146.87 ± 22.28	0.300	0.830	0.334	0.262
NU	109.82 ± 25.97	111.15 ± 21.19	112.15 ± 18.66	0.402	0.961	0.257	0.313
NL	93.45 ± 23.23	96.08 ± 18.92	89.21 ± 17.75	0.678	0.619	0.373	0.179
IN	152.73 ± 40.69	149.62 ± 42.01	157.72 ± 20.07	0.524	0.731	0.541	0.355
IT	155.79 ± 28.64	151.73 ± 31.67	158.21 ± 15.54	0.461	0.541	0.686	0.313
TL	75.61 ± 13.24	80.54 ± 20.40	79.49 ± 18.91	0.977	0.289	0.355	0.815
TU	81.91 ± 14.68	87.00 ± 17.77	84.49 ± 28.13	0.818	0.374	0.617	0.649
ST	136.18 ± 21.39	140.54 ± 30.89	136.41 ± 20.46	0.783	0.489	0.968	0.497

A, active group; I, inactive group; H, health controls, a *p*-value of <0.05 is considered statistically significant.

### 3.3 Correlation between VD and RNFLT in the peripapillary region and ocular parameters

**Figure 5:**
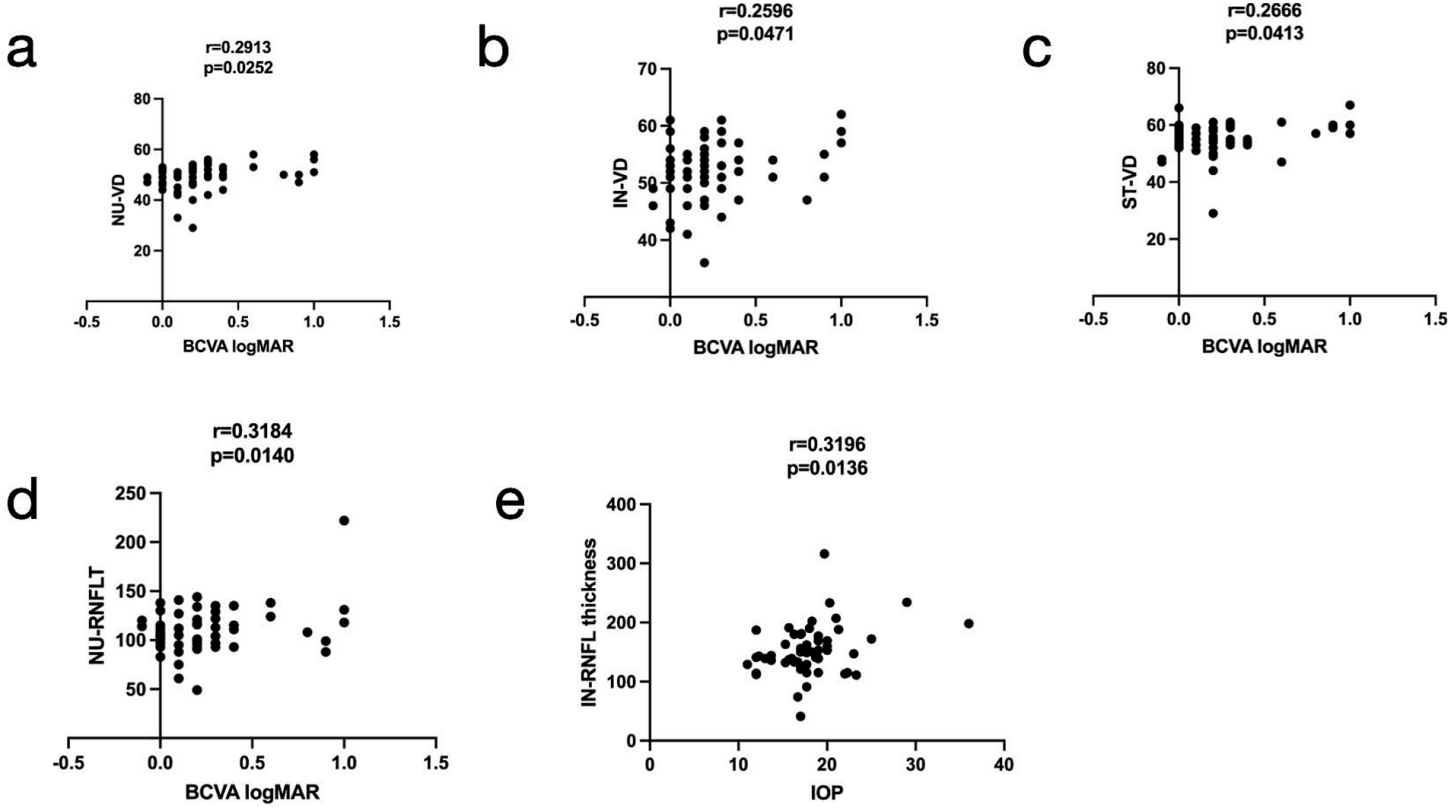
Relationships among the vessel density (VD), retinal nerve fiber layer thickness (RNFLT) in the radial peripapillary region, best-corrected visual acuity (BCVA) logMAR, and intraocular pressure (IOP). The nasal upper (NU)-VD, inferior nasal (IN)-VD, superior nasal (SN)-VD, and NU-RNFLT (**a–d**, respectively) were positively associated with BCVA LogMAR (*r* = 0.2913, 0.2596, 0.2666, and 0.3184, respectively), and the IN-RNFLT **(e)** was positively associated with IOP (*r* = 0.3196).

We analyzed the relationship between VD and RNFLT in the eight sectors of the peripapillary region and their association with the BCVA logMAR, duration, IOP, and TSH levels in the TED group. No significant correlations were found between overall VD and RNFLT in the entire peripapillary region and ocular variations. However, the correlation coefficients of NU-VD, IN-VD, ST-VD, and NU-RNFLT were positively associated with BCVA LogMAR (*r* = 0.2913, 0.2596, 0.2666, and 0.3184, respectively). In addition, a positive correlation was observed between IOP and the IN-RNFLT score (*r* = 0.3319, *p* = 0.014) (Figure 5).

Multiple linear regression analyses showed that none of the risk factors, including sex, duration of hyperthyroidism, smoking status, therapy history, and IOP, were significantly correlated with VD and RNFLT changes.

## 4 Discussion

The etiology of TED as an autoimmune disorder remains unclear, and it manifests with swelling of the eyelids, pain, diplopia, proptosis, redness, and caruncle. Dysthyroid optic neuropathy is a sight-threatening complication of TED. Patients with dysthyroid optic neuropathy may experience permanent visual loss. The pathogenesis of DON involves both mechanical compression from orbital tissue expansion and ischemic insult. Furthermore, orbital venous stasis—a feature observed in both the active and inactive stages of TED—has been strongly implicated in the development of DON (9).

We usually identify the clinical features leading to a diagnosis of DON, such as reduced visual acuity, visual field defects, and optic disc swelling (21). However, clear diagnostic criteria for DON remain to be established, and current diagnosis relies on a combination of clinical features and tests, including visual field (VF), visual evoked potential (VEP), and CT or MRI imaging (22). Huang X et al. revealed that TED was often misdiagnosed, especially in the early stage (23). McKeag et al. (24) reported that normal visual acuity did not preclude the diagnosis; nearly half of the patients had a normal optic disc appearance, and the eyes of many patients with DON were not significantly proptosed. One study (25) revealed that patients with DON may test positive on one test but negative on another. Exploring additional auxiliary tests is crucial for the diagnosis of DON and should be interpreted in correlation with clinical features. Previous studies (26, 27) have shown that color Doppler imaging (CDI), a non-invasive technique, reveals a reduction in SOV blood flow velocity, which correlates with TED severity. However, CDI can be influenced by subjective factors, such as eye movement and pressure on the eyeball (9).

Recently, OCTA has been widely used as a new, non-invasive, detailed, and quantitative technology for studying the microvasculature of retinal and choroidal vascular networks in a 3D manner (28). In this study, we aimed to explore changes in the microvasculature and RNFLT in patients with TED using OCTA, characterize ONH perfusion changes across different TED stages, and evaluate its potential utility as a monitoring auxiliary test, including in DON cases.

Previous studies have demonstrated that vessel density in the peripapillary region differs significantly between eyes with TED and healthy eyes. Zhang et al. (29) reported that the whole-image vessel densities of the RPC and ONH decreased significantly in eyes with DON, particularly in the temporal sectors. Mihailovic et al. (30) evaluated patients with inactive TED and demonstrated reduced VD in the macula and ONH. However, they analyzed only the whole RPC region. Wu et al. (31) divided the RPC region into eight sectors according to the Garway–Heath map (20) and found that the VD in the RPC region was significantly lower in the equivocal DON group than in the healthy group. In this study, we attempted to characterize changes in ONH and RPC perfusion in patients with TED (active and inactive) using OCTA. We found that the peripapillary flow index and VD were reduced in the RPC region (Figure 4), especially in patients with inactive SN and ST sectors. Wang, XN. et al. (32) pointed out that the optic nerve and vessel density in the ST sectors of the optic disc are denser than those in other quadrants, based on anatomical points. They also found that, in the ST area, the peripapillary VD and RNFLT were lower in the early diabetes retinopathy group than in the normal group. Mansoori et al. (17) demonstrated that RPC density was highest in the arcuate region, and chronic inflammatory changes could cause atrophic changes. Furthermore, blood flow was reduced secondary to decreased oxygen consumption, (33) which might explain the result of reduced VD in the SN and ST sectors of patients in the inactive stage. Therefore, early detection of RPC density in these sectors would be essential for indicating TED progression. However, no significant difference in ONH-VD was observed between the active and inactive stages, a finding consistent with a previous report (34). In addition, while differences in RNFLT across all eight sectors were observed between both active and inactive patients compared to HCs, there was no statistical discrepancy, which could be attributed to the small sample size.

Decreased peripapillary VD of the ST, IN, and NU sectors and reduced NU-RNFLT were positively correlated with the extent of visual acuity impairment. The alterations in RPC density and RNFLT in TED were associated with BCVA, even in some sectors. We hypothesize that these results may have a predictive value in monitoring TED using OCTA. Awareness of changes in RPC density alteration should be an important clinical goal for TED management. Furthermore, our study found that the IOP in the active and inactive TED patients was significantly higher than that in the HCs. Konuk, Onur et al. and other studies (10, 35) have revealed that elevated episcleral venous pressure and retrobulbar pressure have been reported as contributing factors to reduced orbital venous drainage, which may also account for increased IOP in patients with TED. IN-RNFLT was positively correlated with IOP in patients with TED. Some studies (31, 34) have shown that hypoxic ischemia and increased orbital tension result from increased IOP, and the increased IOP changes RNFLT.

This study has some limitations. First, the sample size is small. Further studies with larger sample sizes are required to confirm our results. Furthermore, although there was no steroid pulse therapy or local radiotherapy in the last 3 months in the patients with TED, their ultimate effect on our results cannot be completely ruled out, and we should consider separate analyses of treated patients in future studies. Moreover, this was a cross-sectional study, which could not elaborate on the changes in papillary microcirculation at different stages in the same patient with TED. An improvement would be to conduct longitudinal observations to analyze alterations in ONH blood flow in patients with TED throughout disease progression. Finally, while OCTA provides valuable information on ONH perfusion, which can indicate blood flow, it may not accurately capture slower blood flow.

In summary, this study demonstrated that decreased microvasculature can be visualized in patients with TED using OCTA, revealing functional and structural impairments around the optic disc. The measurement based on the ONH topographic map may be more sensitive than traditional methods. Furthermore, additional longitudinal studies are needed to validate the usefulness of OCTA outcomes as a monitoring tool for TED progression.

## Data Availability

The original contributions presented in the study are included in the article/supplementary material, further inquiries can be directed to the corresponding authors.
